# A mutation in budding yeast *BRR6* affecting nuclear envelope insertion of the spindle pole body

**DOI:** 10.17912/micropub.biology.000463

**Published:** 2021-09-16

**Authors:** Jennifer M Gardner, Eileen O'Toole, Sue L Jaspersen

**Affiliations:** 1 Stowers Institute for Medical Research, Kansas City, MO 64110; 2 Department of Molecular, Cellular and Developmental Biology, University of Colorado, Boulder, CO 80309-0347; 3 Department of Molecular and Integrative Physiology, University of Kansas Medical Center, Kansas City, KS 66160

## Abstract

*BRR6* and *BRL1 *are two paralogs that encode transmembrane proteins of the nuclear envelope (NE) involved in membrane fluidity and nuclear pore complex biogenesis in organisms that undergo a closed mitosis. We show that mutation of a conserved cysteine in the intralumenal domain of *Saccharomyces cerevisiae* Brr6p results in a novel temperature sensitive allele, *brr6-Y100H*, that arrests growth due to defects in spindle formation. Analysis of *brr6-Y100H* cells by electron tomography and Brr6p localization by super-resolution imaging supports the idea that Brr6p is involved in insertion of the newly duplicated spindle pole body into the NE.

**Figure 1. Brr6p localization and function at the budding yeast SPB:**
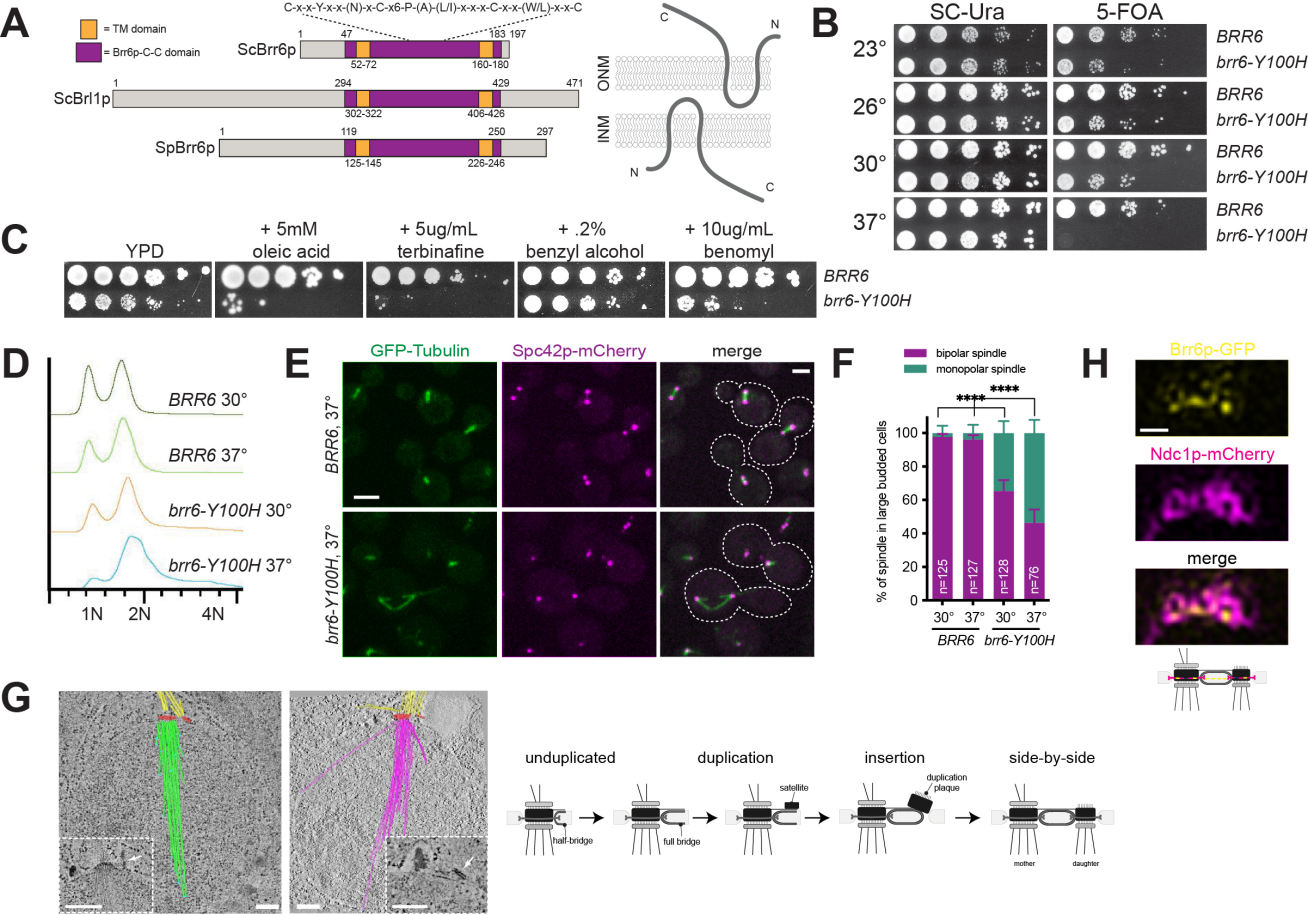
A. Brr6p, Brl1p and *S. pombe* Brr6 domain structure, including the transmembrane domains and the signature Brr6p-C-C domain. Schematic of *S. cerevisiae* Brr6p topology in the NE based on data from (Zhang *et al.* 2018). B. *BRR6* and *brr6-Y100H* containing plasmids were integrated into *brr6∆::KANMX pURA3-BRR6* (SLJ6805) and the ability of each to function at the indicated temperatures was tested by plating 10-fold serial dilutions of cells onto SC-URA or 5-FOA, which selects for cells that have lost the *pURA3-BRR6* covering plasmid. Plates were incubated for 2 d at 30°C and 37°C and for 3 d at 23°C and 26°C. C. *BRR6* (SLJ9382) and *brr6-Y100H* (SLJ9383) cells were assayed for growth for 2 d at 30°C on YPD plates alone or with the indicated chemicals. D-G. Mid-log phase cultures of *BRR6* (SLJ9382) and *brr6-Y100H* (SLJ9383) were grown at 30°C or shifted 37°C for 4 h. D. DNA content was analyzed by flow cytometry. E. Spindle morphology was analyzed by fluorescence microscopy using GFP-Tub1p (green) and Spc42p-mCherry (magenta) to detect microtubules and SPBs, respectively. Images of large-budded *BRR6* and *brr6-Y100H* cells from the 37°C culture are shown with the cell outline (dashed lines) based on the widefield image. Bar, 2 µm. F. The percentage of large-budded cellscontaining bipolar and monopolar spindles is shown for each strain. Error bars, SEM. ****, p<0.0005 based on Student’s t-test. G. Spindle morphology in *brr6-Y100H* cells grown at 37°C was examined by electron tomography. Projections of spindles in two nuclei are depicted with nuclear microtubules highlighted in green (left tomogram; 29 nuclear microtubules) or magenta (right tomogram; 26 nuclear microtubules), cytoplasmic microtubules shown in yellow, microtubule plus ends marked in cyan and the SPBs represented in red. Insets show individual slices with a mature duplication plaque (arrowhead) and that is also colored in red in the tomograms. It is connected to the mother SPB and nucleates cytoplasmic microtubules. Bars, 100 nm. See Videos S1 and S2 for complete tomograms. On the right, a schematic of SPB duplication, including insertion of the new SPB that is blocked in *brr6-Y100H* mutants. H. SIM of Brr6p-GFP (yellow) and Ndc1p-mCherry (magenta) in cells undergoing SPB duplication. Bar, 200 nm. The cartoon depicts the position of both proteins in the side-by-side SPB.

## Description

Nuclei, the defining characteristic of eukaryotic cells, are bound by a double lipid bilayer, referred to as the nuclear envelope (NE). Prior to mitosis, components of the microtubule cytoskeleton including microtubule-organizing centers (known as centrosomes in metazoans and spindle pole bodies (SPBs) in fungi) that are located in the cytoplasm throughout interphase must access replicated sister chromosomes located inside the nucleus to form the bipolar mitotic spindle. In metazoans, the NE breaks down (open mitosis); however, in fungi, SPBs are inserted into the NE and the NE remains intact during mitosis (closed mitosis) (reviewed in (Heath 1980; Makarova and Oliferenko 2016)). How a giant protein complex such as a SPB is inserted into the NE is largely unknown, although in budding yeast SPB insertion into the nuclear membrane is coupled with SPB duplication (Jaspersen 2021; Ruthnick and Schiebel 2018).

*BRR6* encodes an essential transmembrane protein involved in lipid homeostasis, nuclear pore complex (NPC) assembly and transcription regulation in *Saccharomyces cerevisiae* (de Bruyn Kops and Guthrie 2001; Hodge *et al.* 2010; de Bruyn Kops *et al.* 2018; Lone *et al.* 2015; Zhang *et al.* 2018). Analysis of *brr6+* function in fission yeast also suggested a role in SPB insertion into the NE (Tamm *et al.* 2011). Studies of Brr6p localization, binding partners and depletion phenotype (along with its paralog, *BRL1*) in budding yeast indicated a primary role in NPC biogenesis rather than SPB insertion (Zhang *et al.* 2018). Like SPBs, NPCs are multi-subunit protein complexes that are inserted into an intact NE. Genetic analysis in yeast indicates that membrane insertion of both NPCs and SPBs likely share common factors, including proteins involved in membrane curvature and stabilization (Casey *et al.* 2012; Chen *et al.* 2014; Chial *et al.* 1998; Witkin *et al.* 2010).

Previous work showed that Brr6p is a component of the NE, with its termini located in the nucleus and cytoplasm and its conserved central (Brr6p-C-C) domain located in the lumen between the inner nuclear membrane (INM) and outer nuclear membrane (ONM) (Figure 1A) (Zhang *et al.* 2018). The Brr6p-C-C domain is a highly conserved motif in Brr6p family members (Tamm *et al.* 2011), with cysteine residues that form intra- or inter-molecular disulfide bridges (Zhang *et al.* 2018). Mutation of a non-conserved residue in this domain (R110K, *brr6-1*) resulted in a conditional growth defect (de Bruyn Kops and Guthrie 2001), and mutation of all four cysteine residues resulted in a lethal allele (Zhang *et al.* 2018).

To further explore the role of the Brr6p-C-C domain in Brr6p function, we created a series of mutations within conserved residues and assayed complementation of *brr6∆* using a plasmid shuffle strategy to determine the functionality of each construct. The ability to grow on 5-fluoroorotic acid (5-FOA) indicates the plasmid containing a wild-type copy of *BRR6* can be lost and the deletion allele is able to rescue all essential Brr6p functions. We found that mutation of the conserved tyrosine located in the Brr6p-C-C domain to histidine (*brr6-Y100H*) resulted in reduced growth compared to wild-type at 23°, 26° and 30°C (Figure 1B). At 37°C, *brr6-Y100H* mutants failed to grow, indicating that it is a temperature sensitive allele of *BRR6*. Interestingly, mutation of the same conserved tyrosine in the Brr6p paralog Brl1p to histidine (*brl1-Y347H*) had no obvious growth defect at any temperature, and cellular morphology appeared normal.

Similar to *brr6-1* mutants (Hodge *et al.* 2010), we found that addition of the sterol biosynthesis inhibitor terbinafine exacerbated the growth defect of *brr6-Y100H* cells at 30°C (Figure 1C), suggesting that defects in the Brr6p-C-C domain result in accumulation of ergosterol biosynthesis intermediates. However, *brr6-Y100H* mutants were only sensitive to addition of the unsaturated fatty acid oleic acid and not to the membrane fluidizer benzyl alcohol, consistent with the idea that loss of Brr6p function does not result in a growth arrest due to an inability to adapt to membrane perturbations (Figure 1C). We also found that growth of *brr6-Y100H* cells was exacerbated by addition of the microtubule depolymerizing agent benomyl (Figure 1C). Benomyl sensitivity is frequently observed in yeast mutants affecting mitotic spindle assembly.

To examine spindle structure, wild-type (*BRR6*) and mutant (*brr6-Y100H*) cells expressing *GFP-TUB1* and *SPC42-mCherry* were grown overnight at the permissive temperature of 30°C then shifted to the non-permissive temperature of 37°C for 4 h. Flow cytometric analysis of DNA content revealed that *brr6-Y100H* cells shifted to 37°C accumulated with a 2N DNA content (Figure 1D). This suggests that *brr6-Y100H* cells arrest in mitosis at the restrictive temperature. Examination of microtubule structures showed that in large budded wild-type cells at both 30° and 37°C, virtually all spindles were bipolar with two foci of Spc42p-mCherry connected by GFP-Tub1p (Figure 1E-F). A fraction of *brr6-Y100H* mutants form bipolar spindles, however, 35% and 54% of large budded *brr6-Y100H* mutants at 30°C and 37°C, respectively, had monopolar spindles: a single focus of Spc42p-mCherry associated with GFP-Tub1p (Figure 1E-F). In many cases, a second SPB signal could be detected in *brr6-Y100H* mutants, as shown in the upper cell in Figure 1E. This ‘dead’ pole is thought to arise from defects in NE insertion of the new SPB during SPB duplication, while insertion of the mother SPB in the previous cell cycle is unaffected. This phenotype has been reported in mutants in *NDC1*, *NBP1*, *BBP1*, *MPS1*, *MPS2* and *MPS3*, which play roles in nascent SPB assembly into the NE (reviewed in (Ruthnick and Schiebel 2018)).

Using electron tomography, we reconstructed spindles from ten *brr6-Y100H* mutant nuclei in cells grown at 37°C. Four nuclei contained two SPBs that had both inserted into the NE to result in duplicated side-by-side SPBs (2 nuclei), metaphase (1 nucleus) or anaphase (1 nucleus) spindles. In the remaining six nuclei, a distinct type of monopolar spindle was observed (Figure 1G, Video S1 & S2): SPB duplication had occurred, the mother SPB was located in the NE but insertion of the nascent SPB into NE was blocked, often resulting in a steep angle between this pole and the nuclear membrane. The newly formed SPB matured to a point that it, like the mother SPB, was able to nucleate cytoplasmic microtubules. However, because of the defect in NE insertion, the new SPB is unable to nucleate nuclear microtubules (hence, it is a ‘dead’ pole). Interestingly, the number of cytoplasmic microtubules formed at the mother SPB was high compared to a typical haploid SPB. Part of this may be due to increased SPB size compared to wild-type cells. The number of nuclear microtubules also increased to 37-56 microtubules/SPB compared to the typical 18-22 seen in wild-type haploids. Moreover, nuclear microtubules were long, often extending across the entire nucleus (Figure 1G).

Our observation that *brr6-Y100H* arrests in mitosis after a 4 h shift to the non-permissive temperature, combined with our cytological analysis demonstrating that over half of these cells contain nuclei with monopolar spindles, points to a requirement for Brr6p in insertion of the new SPB into the NE. Although *brr6-Y100H* cells have other phenotypes including those observed in *brr6-1* and *brr6^dg^* (Hodge *et al.* 2010; Zhang *et al.* 2018), such as a herniations associated with NPC assembly defects, overproliferation of the NE, accumulation of lipid droplets and defects in mitochondrial morphology, these defects are typically not associated with a first or second cell cycle arrest. Most NPC assembly mutants also do not delay in mitosis due to spindle/SPB duplication errors. Consistent with the idea that lethality is independent of NPC assembly, we found that transport of a variety of cargos into and out of the nucleus was unaffected by in *brr6-Y100H* mutants.

In fission yeast, Brr6p transiently localizes to the SPB during insertion into the NE (Tamm *et al.* 2011). Brr6p enrichment at the SPB has not been reported in budding yeast (Zhang *et al.* 2018), although if it is targeted to the SPB during insertion, which occurs during a brief window of the cell cycle, SPB localization may have been overlooked. Using structured-illumination microscopy (SIM), Ndc1p-mCherry can be visualized as a ring-like structure that is linked to SPB insertion into the NE (Chen *et al.* 2019). Brr6p-GFP co-localized with Ndc1p-mCherry in cells that contained duplicated side-by-side SPBs that just completed SPB duplication (Figure 1H).

Our data showing the Brr6p localizes to the SPB, in combination with our analysis of *brr6-Y100H* reporting a defect in nascent SPB insertion into the NE, supports a role for the protein at the SPB in budding yeast. Previous work on Brr6p showed interactions with NPC components as well as with reticulons involved in membrane curvature (Zhang *et al.* 2018). Its SPB localization and function in SPB insertion could be mediated by the NPC itself, which is found near the site of SPB insertion (Ruthnick *et al.* 2017) or interactions between Brr6p and NPC components, including the dual SPB and NPC component Ndc1p that binds to Brr6p (Zhang *et al.* 2018). Interestingly, we did not detect Brl1p at the SPB, and the equivalent mutation in its Brr6p-C-C domain had no effect on growth, indicating that the role for Brr6p at the SPB is specific to it and not a function shared with its paralog. At a mechanistic level, the function of Brr6p in SPB and/or NPC insertion into the NE is unknown, although our work showing defects in *brr6-Y100H* mutants suggests that the conserved Brr6p-C-C domain may play a key role. Formation of disulfide bonds and higher-order folding of this domain could facilitate the membrane curvature needed for SPB and NPC insertion. However, the Brr6p-C-C domain could also function to stabilize Brr6p protein. Consistent with this later idea, the *brr6-Y100H* allele is not viable when present in single copy; the temperature sensitive strains studied here contain two or four copies of the mutant gene. Future analysis of Brr6p binding partners will help address the role that it plays in both SPB and NPC insertion into the NE.

## Methods

*Yeast Strains and Plasmids*

All strains are derivatives of W303 (*ade2-1 trp1-1 leu2-3,112 ura3-1 his3-11,15 can1-100 RAD5+*) and are listed in Reagents along with plasmids used in this study. Standard techniques were used for DNA and yeast manipulations. Tagging and deletion of genes was also done by PCR-based methods (Longtine *et al.* 1998; Sheff and Thorn 2004). ~700 bp of promoter, ~200 bp of terminator and the *BRR6* open-reading frames were amplified by PCR from genomic DNA and cloned into pRS316 and pRS303-based plasmids (Sikorski and Hieter 1989). Mutations in *BRR6*were introduced using the QuikChange II Mutagenesis kit (Agilent) and confirmed by sequencing. Diploid yeast containing *brr6∆::KANMX* were transformed with p*URA3-BRR6* (pSJ1587), sporulated and dissected to create strains used for plasmid-shuffle. pRS303-based plasmids were digested with NheI and integrated into the *HIS3*locus. To analyze phenotypes, cells were cultured overnight in YPD (1% yeast extract, 2% peptone, 2% glucose) then plated to synthetic complete media lacking uracil (SC-Ura) or 5-fluoro-orotic acid (5-FOA) media and grown at the indicated temperatures. Chemicals were purchased from Sigma-Aldrich and were added to YPD media in the following final amounts: 5 mM oleic acid, 5 µg/ml terbinafine, 2% benzyl alcohol or 10 µg/ml benomyl.

*Cell Growth, Flow Cytometry and Confocal Imaging*

Single colonies picked from 5-FOA plates were grown overnight to mid-log phase at 30°C in YPD, then kept at 30°C or shifted to 37°C for 4 h before harvesting and analysis. DNA content was analyzed by flow cytometry in sonicated cells that had been fixed with 70% ethanol for 1 h at room temperature, treated with RNAse (Roche) and Proteinase K (Roche) for 2 h to overnight at 37°C and stained with propidium iodide (Sigma-Aldrich) in the dark at 4°C overnight. Samples were analyzed on a MACSQuant FACS Analyzer (Miltenyi Biotec) and data was displayed using FlowJo software (Tree Star). Spindle structure was assayed by live cell imaging on a Perkin Elmer Ultraview spinning disk confocal microscope equipped with a Hamamatsu EMCCD (C9100-13) optimized for speed, sensitivity and resolution. The microscope base was a Carl Zeiss Axio-observer equipped with an αPlan-Apochromat 100x 1.46NA oil immersion objective and a multiband dichroic reflecting 488 and 561 nm laser lines. GFP images were acquired with 488 nm excitation and 500-550 nm emission. mCherry images were acquired with 561 nm excitation and 580-650 nm emission. Data were acquired using the Perkin Elmer Volocity software with a z-spacing of 0.4 µm. Exposure time, laser power and camera gain were maintained at a constant level chosen to provide high signal-to-noise but avoid signal saturation for all samples. Images were processed using Image J (NIH). A representative z slice image is shown.

*Structured-Illumination Microscopy*

Cells were fixed for 15 minutes in 4% paraformaldehyde (Ted Pella) in 100 mM sucrose, then washed two times in phosphate-buffered saline, pH 7.4. An aliquot of cells was placed on a glass slide and covered with a number 1.5 coverslip. SIM images were acquired with an Applied Precision OMX Blaze (GE Healthcare). A 60X 1.42 NA Plan Apo oil objective was used, and emission was collected onto two PCO Edge sCMOS cameras with each camera dedicated to one specific channel. For the two-color GFP/mCherry experiments, a 405/488/561/640 dichroic was used with 504- to 552-nm and 590- to 628-nm emission filters for GFP and mCherry, respectively. Images were taken using a 488-nm laser (for GFP) or a 561-nm laser (for mCherry), with alternating excitation. SIM reconstruction was performed with the Applied Precision software SoftWoRx with a Wiener filter of 0.001. Color alignment from different cameras in the radial plane was performed using the color alignment slide from GE Healthcare. In the axial direction, color alignment was performed using 100 nm TetraSpeck beads (Thermo FIsher). For image preparation, the SIM reconstructed images were scaled 2×2 with bilinear interpolation in ImageJ, then smoothed with a Gaussian blur of pixel radius 0.8.

*Transmission Electron Microscopy*

*brr6-Y100H* cells were grown overnight at 30°C and then shifted into a pre-warmed 37°C water bath for 4 h. Cells were quickly harvested and frozen on the Leica EM-Pact at ~2050 bar, transferred under liquid nitrogen into 2% osmium tetroxide/0.1% uranyl acetate/acetone, and transferred to the Leica AFS. The freeze substitution protocol was as follows: −90° for 16 h, raised 4°/h for 7 h, −60° for 19 h, raised 4°/h for 10 h, and −20° for 20 h. Samples were then removed from the AFS, placed in the refrigerator for 4 h, and then allowed to incubate at room temperature for 1 h. Samples went through three changes of acetone over 1 h and were removed from the planchettes. They were embedded in acetone/Epon mixtures to final 100% Epon over several days in a stepwise procedure as described (McDonald 1999). Sixty-nanometer serial thin sections were cut on a Leica UC6, stained with uranyl acetate and Sato’s lead and imaged on a FEI Tecnai Spirit.

*Electron Tomography*

Tomography was performed as previously described (Giddings *et al.* 2001). Dual-axis tilt series data were collected on a FEI Tecnai F20 at 200 kV. SerialEM (Mastronarde 2005) was used to acquire images every 1° over a ±60° range on a Gatan CCD camera with a pixel size of 1-1.5 nm. Images were aligned to compute tomograms using the IMOD software package (Mastronarde 1997). Nuclei spanned two to five serial sections, which were joined to produce the final volume shown. Ten nuclei were examined, including two nuclei with duplicated side-by-side SPBs, one nucleus with a metaphase spindle and another with an anaphase spindle. In the remaining six nuclei, monopolar spindles similar to those shown in the Figure were observed. The tomograms were displayed and modeled using the 3dmod program in IMOD, including labeling each microtubule, microtubule end and the SPB. Supplemental videos show example raw EM images and nuclear reconstructions from these images.

## Reagents

**Table:** 

**Yeast Strains**		
Strain	Genotype	Figure
SLJ6805	*MATa brr6∆::KANMX pURA3-BRR6*	Figure 1B
SLJ9382	*MATa brr6∆::KANMX his3::BRR6-HIS3MX pURA3-BRR6 ADE2 LYS2*	Figure 1C
SLJ9383	*MATa brr6∆::KANMX his3::brr6-Y100H-HIS3MX pURA3-BRR6 ADE2 LYS2*	Figure 1C
SLJ6994	*MATa brr6Δ::KANMX his3::BRR6-HIS3 trp1::GFP-TUB1-TRP1 pLEU2-SPC42-mCherry-HIS3MX ADE2*	Figure 1D-F
SLJ6995	*MATa brr6Δ::KANMX his3::brr6-Y100H-HIS3 trp1::GFP-TUB1-TRP1 pLEU2-SPC42-mCherry-HIS3MX ADE2*	Figure 1D-F
SLJ6832	*MATa brr6∆::KANMX his3::brr6-Y100H-HIS3*	Figure 1G
SLJ10077	*MATa BRR6-GFP-HIS3MX NDC1-mCherry-KANMX*	Figure 1H
		
**Plasmids**		
Number	Name	Source
pSJ1587	pRS316-*BRR6*	this study
pSJ1377	pRS303-*BRR6*	this study
pSJ1431	pRS303-*brr6-Y100H*	this study
pSJ906	pRS315-*SPC42-mCherry-HIS3MX*	Rong Li Lab
